# Quantitative comparison of left ventricular motion parameters for the assessment of asynchrony and motion abnormalities

**DOI:** 10.1186/1532-429X-16-S1-P94

**Published:** 2014-01-16

**Authors:** Raphael Beck, Jan Paul, Anja Müller-Lutz, Dominik Buckert, Peter Bernhardt, Wolfgang Rottbauer, Volker Rasche

**Affiliations:** 1 Internal Medicine II, University Hospital of Ulm, Ulm, Germany; 2 Department of Diagnostic and Interventional Radiology, University of Dusseldorf, Medical Faculty, Dusseldorf, Germany

## Background

Different motion parameters for quantification of heart motion abnormalities and asynchrony have been described in literature. The aim of this study is the direct comparison of reported parameters in different patient groups.

## Methods

Cohorts and Acquisition: 41 volunteers (HV, 25 ± 5 y.), 14 STEMI (63 ± 7 y.), 12 DCM (54 ± 17 y.), and 5 DCM+LBBB (47 ± 8 y.) patients were investigated. Acquisition parameters were: Philips Achieva 3 T, 32 channel cardiac coil, velocity encoded (Tissue Phase Mapping, TPM) segmented black-blood gradient echo with VENC = 30 cm/s, TR/TE = 6.1/4.6 ms, FOV adapted to patient size, resolution = 2^2^x8 mm^3^, 3 k-lines/segment, SENSE = 2, phase interval = 30 ms, and nominal scan time = 5:51 min:sec for 3 short axis slices. Parameters (see [1]): a) velocity-based: Standard Deviation of Times to Peak [SD(TTP)], Asynchrony Correlation Coefficient [ACC], Temporal Uniformity of Velocity [TUV], and Velocity Ranges (difference: maximum-minimum velocity). b) strain-based: Base Apex Rotation Correlation [BARC], Temporal Uniformity of Strain [TUS], Standard Deviation of Onset/Peak Time [SD(T)], Coefficient of Variation [CV], Difference between Septal and Lateral Peak Circumferential Strain [DiffSLpeakCS], Onset/Peak Of Shortening Delay [Delay], Regional Variance of Strain [RVS], and Regional Variance Vector of Strain [RVVPS]. Analysis: The significance of resulting differences between the different groups was assessed via non-parametric Kruskal-Wallis-Test.

## Results

**Figure 1:**
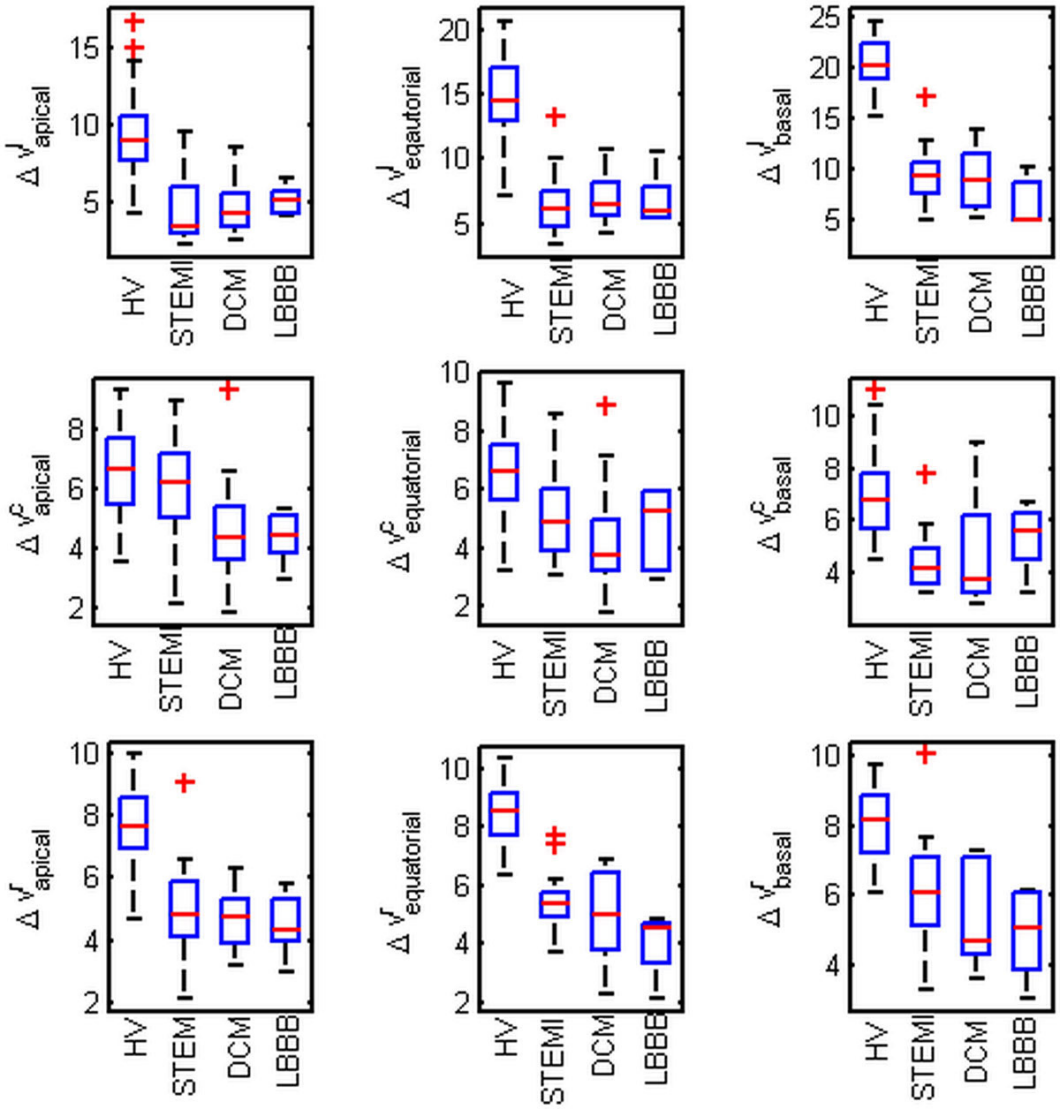
**Distribution of velocity ranges (Δv = v_max _- v_min_) in radial (r), circumferential (c) and longitudinal (l) direction for apical, equatorial and basal slices**. Ranges are decreased in all patient cohorts compared to volunteers.

Statistics of comparison between the different cohorts are presented in table 1. Velocity-based: Velocity ranges are significantly reduced in HV and patients with structural heart disease, especially in radial and longitudinal direction over all three short-axis slices (see Figure 1). SD(TTP) for diastolic, longitudinal velocity is increased in all patients; SD(TTP) for systolic, radial velocity is significantly higher in patients with DCM+LBBB. ACC(longitudinal) is significantly decreased in patient cohorts compared to HV. ACC(radial, average/maximum) separates LBBB from all other cohorts. Strain-based: BARC highlights loss of torsion in patients with DCM and LBBB. TUS, RVS, and RVVPS are able to detect dyssynchrony in LBBB. SD(T, peak) is raised in patients. SD(T, onset) indicates asynchrony in LBBB patients. CV shows significant increase in LBBB vs. all other cohorts. In this study DiffSLpeakCS is not capable to make distinction between HV and patients. Delay(peak, septal-lateral/inferior-anterior) show characteristic aberrations for STEMI and LBBB compared to HV.

**Table 1:** Comparison of parameter differences between different cohorts: not significant (-), p < 5 % (*), p < 1 % (**), p < 0.1 % (***).

Parameter					**HV vs**.			**LBBB vs**.	
					**STEMI**	**DCM**	**LBBB**	**STEMI**	**DCM**

velocity based	.	o	SD(TTP)	systolic, radial diastolic, radial systolic, longitudinal diastolic, longitudinal	- - ** ***	- - - ***	*** ** * ***	** - - *	** - - *

	/	--	ACC	radial, minimum radial, average radial, maximum circumferential, minimum circumferential, average circumferential, maximum longitudinal, minimum longitudinal, average longitudinal, maximum	* - - - - * ** *** **	* * - ** * - * *** ***	*** *** *** * * - ** *** ***	- ** ** - * - - * *	* *** *** - - - - ** **

	/	--	TUV	radial circumferential longitudinal	- - ***	- * *	*** * ***	** - **	* - *

	/		Velocity Range	longitudinal, apical longitudinal, equatorial longitudinal, basal circumferential, apical circumferential, equatorial circumferential, basal radial, apical radial, equatorial radial, basal	*** *** *** - *** *** *** *** ***	*** *** *** *** *** ** *** *** ***	*** *** *** ** * - *** *** ***	- - - * - - - * -	- - - - - - - - -

position/strain based	/	|	BARC		-	-	**	*	*

	/	--	TUS	circumferential radial	- -	- -	*** **	** **	** **

	/	--	RVS		-	*	-	*	***

	/	--	RVVPS		-	-	**	**	***

	.	o	SD(T)	onset	-	*	**	**	*

				peak	***	***	***	-	*

	.	o	CV		-	-	***	**	**

	.	--	DiffSLpeakCS		-	-	-	-	-

	.	-- -- | -- -- |	Delay	onset, septal-lateral onset, inferior-anterior onset, apical-basal peak, septal-lateral peak, inferior-anterior peak, apical-basal	- - - ** *** *	* - ** - - -	* - - *** *** **	- - - * - -	- - - * * -

Parameters are categorized by calculation on whole time course (/) vs. single time points (.), and by calculation within each slice (--), between different slices (|), or over the entire left ventricle (o).

## Conclusions

Most distinct results were found in velocity ranges, indicating damaged myocardium, although they featured no particular value for differentiation between LBBB and other patient cohorts. Parameters calculated on the entire cardiac cycle in each slice (velocity and strain: ACC, TUS, RVS, RVVPS) proved capable of detecting ventricular asynchrony due to LBBB

## Funding

None.
